# Transfer learning approach in pre-treatment CT images to predict therapeutic response in advanced malignant pleural mesothelioma

**DOI:** 10.3389/fonc.2024.1432188

**Published:** 2024-09-16

**Authors:** Annarita Fanizzi, Annamaria Catino, Samantha Bove, Maria Colomba Comes, Michele Montrone, Angela Sicolo, Rahel Signorile, Pia Perrotti, Pamela Pizzutilo, Domenico Galetta, Raffaella Massafra

**Affiliations:** ^1^ Laboratorio di Biostatistica e Bioinformatica, Istituto di Ricovero e Cura a Carattere Scientifico (IRCCS) Istituto Tumori ‘Giovanni Paolo II’, Bari, Italy; ^2^ Struttura Semplice Dipartimentale di Oncologia Medica Toracica, Istituto di Ricovero e Cura a Carattere Scientifico (IRCCS) Istituto Tumori ‘Giovanni Paolo II’, Bari, Italy

**Keywords:** malignant pleural mesothelioma, convolutional neural network, machine learning, mRECIST, CT images, response to therapy

## Abstract

**Introduction:**

Malignant pleural mesothelioma (MPM) is a poor-prognosis disease. Owing to the recent availability of new therapeutic options, there is a need to better assess prognosis. The initial clinical response could represent a useful parameter.

**Methods:**

We proposed a transfer learning approach to predict an initial treatment response starting from baseline CT scans of patients with advanced/unresectable MPM undergoing first-line systemic therapy. The therapeutic response has been assessed according to the mRECIST criteria by CT scan at baseline and after two to three treatment cycles. We used three slices of baseline CT scan as input to the pre-trained convolutional neural network as a radiomic feature extractor. We identified a feature subset through a double feature selection procedure to train a binary SVM classifier to discriminate responders (partial response) from non-responders (stable or disease progression).

**Results:**

The performance of the prediction classifiers was evaluated with an 80:20 hold-out validation scheme. We have evaluated how the developed model was robust to variations in the slices selected by the radiologist. In our dataset, 25 patients showed an initial partial response, whereas 13 patients showed progressive or stable disease. On the independent test, the proposed model achieved a median AUC and accuracy of 86.67% and 87.50%, respectively.

**Conclusions:**

The proposed model has shown high performance even by varying the reference slices. Novel tools could help to improve the prognostic assessment of patients with MPM and to better identify subgroups of patients with different therapeutic responsiveness.

## 1 Introduction

Malignant pleural mesothelioma (MPM) is a poor-prognosis disease, mainly correlated to asbestos exposure (1–7). In most patients, the diagnosis of MPM occurs at an advanced stage, and systemic chemotherapy is the frontline standard of care even if usually with a palliative intent. New therapeutic strategies, including immune checkpoint inhibitors (ICIs) and antiangiogenetic agents, have shown encouraging results. First-line therapies generally achieve a partial regression or stable disease lasting an average of 6 months and, in 15%–20% of cases, even 1 year (8–14).

The therapeutic response of MPM depends on multiple factors, but the identification of subgroups of patients with different prognoses and responsivity to therapies is still an unmet need (15, 16). Because of the recent availability of new therapeutic options, a better prognostic assessment and the search for biomarkers capable of predicting the response to treatments are increasingly needed. As a further matter, the initial clinical response could represent a parameter useful to identify patients with a better outcome (8, 17).

The last decade has added important insights for a more accurate definition of this heterogeneous disease; both for a correct staging and for a more accurate and updated assessment of the clinical–radiological response, researchers need to make the most of the characteristics of nuclear medicine and radiological imaging for better management of these patients (18–20).

Currently, in clinical practice, the CT scan represents the baseline diagnostic tool available to the multidisciplinary team that provides crucial radiological information in the decision-making process of care.

The hypothesis that motivated this work is that there are specific patient characteristics, particularly quantitative characteristics extracted from pre-treatment radiological images, that can be informative about the early response to therapy. Morphological, morphometric, and textural characteristics not visible to the human eye, but quantifiable through innovative biomedical image analysis techniques using artificial intelligence (AI) techniques, could help guide the clinicians’ therapeutic decision-making.

Indeed, the predictive value of a radiomic signature extracted from radiological images is well established at the state of the art for different oncological settings (21–26). Lung cancer imaging has been recently studied with AI techniques in several settings, focusing on the early detection of pulmonary nodules, histological diagnosis and prognostic assessment, and the prediction of treatment response.

In addition, in patients with MPM, this approach (including radiological and nuclear medicine techniques, such as PET and CT images) has been proposed to provide useful information about the diagnosis and outcome of patients (26–29).

To the best of our knowledge, literature data about radiomic approaches for predicting response to therapy in patients with advanced mesothelioma are scarce. For this reason, AI approaches for a better definition of prognostic models assume an important role in modern medicine in general, and even more in rare pathologies such as the main focus of this work.

In our previous study, we evaluated the prognostic power of clinical factors in predicting the initial therapeutic response of patients with advanced pleural mesothelioma (30); the results have shown that the information power contained in the clinical features alone is not negligible to predicting the initial response to therapy. However, the clinical features alone did not allow us to develop a support instrument suitable for actual clinical application. Here, we have hypothesized that a radiomic signature obtained from CT images of patients with MPM could predict the initial response to the treatment. Accordingly, the radiomic characterization of CT images of target lesions at baseline and at the time of the first disease re-evaluation could represent a possible criterion for discriminating subgroups of patients based on different responsiveness to treatments. Indeed, as already suggested in other neoplastic diseases, this radiomic signature could play a role as an imaging biomarker correlated to the patient’s outcome (31–35).

In the present study, we applied a transfer learning approach on baseline CT scans of patients with advanced/unresectable MPM with the aim to evaluate the ability to early predict the treatment response on the basis of a pre-trained convolutional neural network (CNN).

Pre-trained CNNs refer to a transfer learning technique (36–39): the networks have been previously trained (pre-trained CNNs) on a huge number (millions) of natural non-medical images to learn how to automatically extract features of different levels of abstraction. The acquired knowledge during training is transferred and applied to never unseen images across diverse research fields (transfer learning), such as CT images, to solve a particular task.

Pre-trained CNNs have already been successfully applied to medical imaging to solve lung cancer detection, classification, or diagnosis tasks (39–44). However, such a proposal represents the first effort towards the designing of a support tool to better guide the treatment planning in patients with MPM.

We have selected three slices of baseline CT scan as input to pre-trained CNN to automatically extract low-level features, i.e., related to the local structure of the image, thus overcoming manual feature extraction. Next, an optimal feature set was detected and used to train a support vector machine (SVM) classifier (45) to discriminate responders (partial response) from non-responder (stable or disease progression) patients. Such a model could allow for the early identification of non-responder patients, thus potentially representing a suitable guide for the choice of the treatment plan.

## 2 Materials and methods

### 2.1 Materials

From July 2017 to October 2021, we collected clinical data and CT scan images of 38 consecutive patients affected by advanced/unresectable pleural mesothelioma undergoing first-line systemic treatment. Patients were enrolled according to the following criteria:

- histological diagnosis of MPM,
- first-line systemic treatment for advanced/unresectable disease,
- evaluation after at least two cycles of treatment, and
- availability of the pre-treatment CT scan.

The population sample used in this study is a subsample used in a previous study (30) for which baseline CTs were available.

The study was approved by the Ethic Committee of IRCCS—Istituto Tumori “Giovanni Paolo II” Bari, Italy (Deliberation n. 506/2023). Patients’ characteristics, including the type of treatment, are shown in **Table 1**. The initial response to treatment was evaluated by both clinical examination and CT scan with the mRECIST criteria (18, 19) at baseline and after two to three treatment cycles. For each patient, we have collected pre-treatment CT images that were acquired by baseline CT scans with a thickness of 0.625–3 mm and an x-ray tube current at 124–699 mA at 80–140 kVp.

**Table 1:** Patient characteristics.

Characteristics	Distribution
Initial therapy response	
Partial response (Abs.; %)	25 (65.79%)
Stable or progressive disease (Abs.; %)	13 (34.21%)
Age at diagnosis	
Median (1st–3rd quartiles)	70.27 (69.05–75.60)
Gender	
Female (Abs.; %)	13 (34.21%)
Male (Abs.; %)	25 (65.79%)
Comorbidity	
<1 (Abs.; %)	9 (23.68%)
>1 (Abs.; %)	17 (44.74%)
Nan (Abs.; %)	12 (31.58%)
Asbestos exposure	
Yes (Abs.; %)	12 (31.58%)
No (Abs.; %)	9 (23.68%)
Nan (Abs.; %)	17 (44.74%)
Smoking habit	
Yes (Abs.; %)	8 (21.05%)
No (Abs.; %)	12 (31.58%)
Ex (Abs.; %)	5 (13.16%)
Nan (Abs.; %)	13 (34.21%)
Pack/year	
≤31	17 (44.74%)
>31	8 (21.05%)
Nan (Abs.; %)	13 (34.21%)
Histology	
Non-epithelioid/biphasic (Abs.; %)	9 (23.68%)
Epithelioid (Abs.; %)	29 (76.31%)
Disease stage	
I	0 (-)
II	7 (18.42%)
III	28 (73.69%)
IV	3 (7.89%)
Pleural effusion	
Yes (Abs.; %)	9 (23.68%)
No (Abs.; %)	14 (36.84%)
Nan (Abs.; %)	15 (39.47%)
ECOG	
0–1 (Abs.; %)	28 (73.68%)
2 (Abs.; %)	10 (26.32%)
BMI	
Overweight (Abs.; %)	14 (36.84%)
Normal (Abs.; %)	12 (31.58%)
Nan (Abs.; %)	12 (31.58%)
Systemic therapy	
Ipi-nivo (Abs.; %)	9 (23.68%)
Platinum-pem (Abs.; %)	15 (39.47%)
Platinum-pem-beva (Abs.; %)	7 (18.42%)
Platinum-pem-beva-atezo (Abs.; %)	5 (13.16%)
Platinum-pem-pembro (Abs.; %)	2 (5.27%)

Ipilimumab (Ipi); Nivolumab (Nivo); Pemetrexed (Pem); Bevacizumab (Beva); Pembrolizumab (Pembro); Atezolizumab (Atezo). Not Available (NA); Body Mass Index (BMI).

### 2.2 Radiomic feature extraction by pre-trained CNN

**Figure 1:**
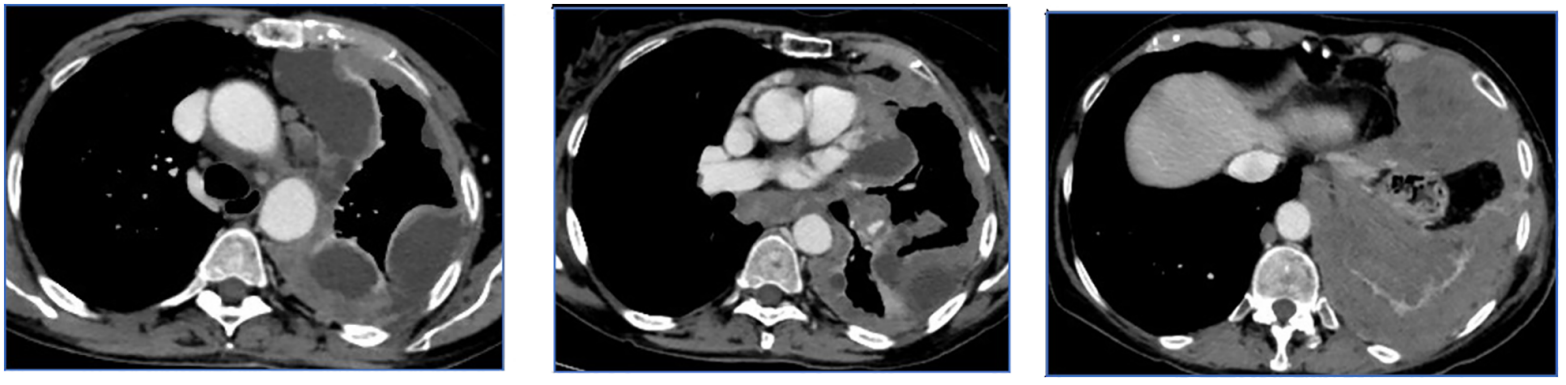
Example of slices selected according to the mRECIST criteria. The baseline CT scan was evaluated by the portal phase study after contrast medium injection, which allows the best visualization of the pleura. On the basis of the slices selected by the radiologist, radiomic features are extracted from the convolutional neural network and subsequently processed with machine learning techniques.

All CT images, at baseline and after two to three treatment cycles, have been retrospectively selected and evaluated by an expert radiologist with more than 20 years of experience. For each patient, measurements at three levels of the pleural lesions were made, and three CT slices were selected, including the target lesions according to the mRECIST criteria (**Figure 1**). The CT scans were evaluated through the portal phase study after contrast medium injection, which allows the best visualization of the pleura in all its extension.

Given the recent success of the so-called transfer learning approach across disparate fields of medical application, we used a high-performing pre-trained CNN architecture, called AlexNet, as a feature extractor (36–39). The transfer learning approach is used when the sample studied is small to circumvent the data requirement for the training process of a deep neural network; it consists of extracting radiomic features by using a pre-training CNN on millions of images of different natures. The knowledge learned by the network was here transferred to our images to fulfill our classification task. AlexNet is a CNN with eight deep layers (36); it has a hierarchical structure where deeper levels are built using the functionality of the previous level. AlexNet was previously trained on millions of images of different kinds to solve discrimination tasks; the data obtained were transferred to our images to extract features useful to train a classification model to predict the treatment response.

AlexNet needs an image input size of 227 × 227; therefore, each CT slice was first resized to patches of this size. The radiomic features were extracted from planning DICOM files. We have extracted features from the first pooling layer (called “pool2”) that had an output with a size of 13 × 13 × 256 that was flattened to a single 129,792-length feature vector. The extracted features are low-level features, i.e., local details of the image, such as edges, dots, and curves.

For each patient, three slices of the baseline CT scan were used as input to the pre-trained AlexNet. Therefore, three radiomic feature vectors were associated with each case. To obtain a single vector radiomic feature corresponding to each individual patient, we calculated the average value of each feature. Since the selection of the three slices of interest, used to assess the disease extent, is operator-dependent, in this study, we aimed to verify how robust the developed model was when subjected to variations of the analyzed slices. Consequently, we evaluated the classification performance by shifting randomly two slices up and down, with respect to those selected by our operator.

### 2.3 Classification model design

**Figure 2:**
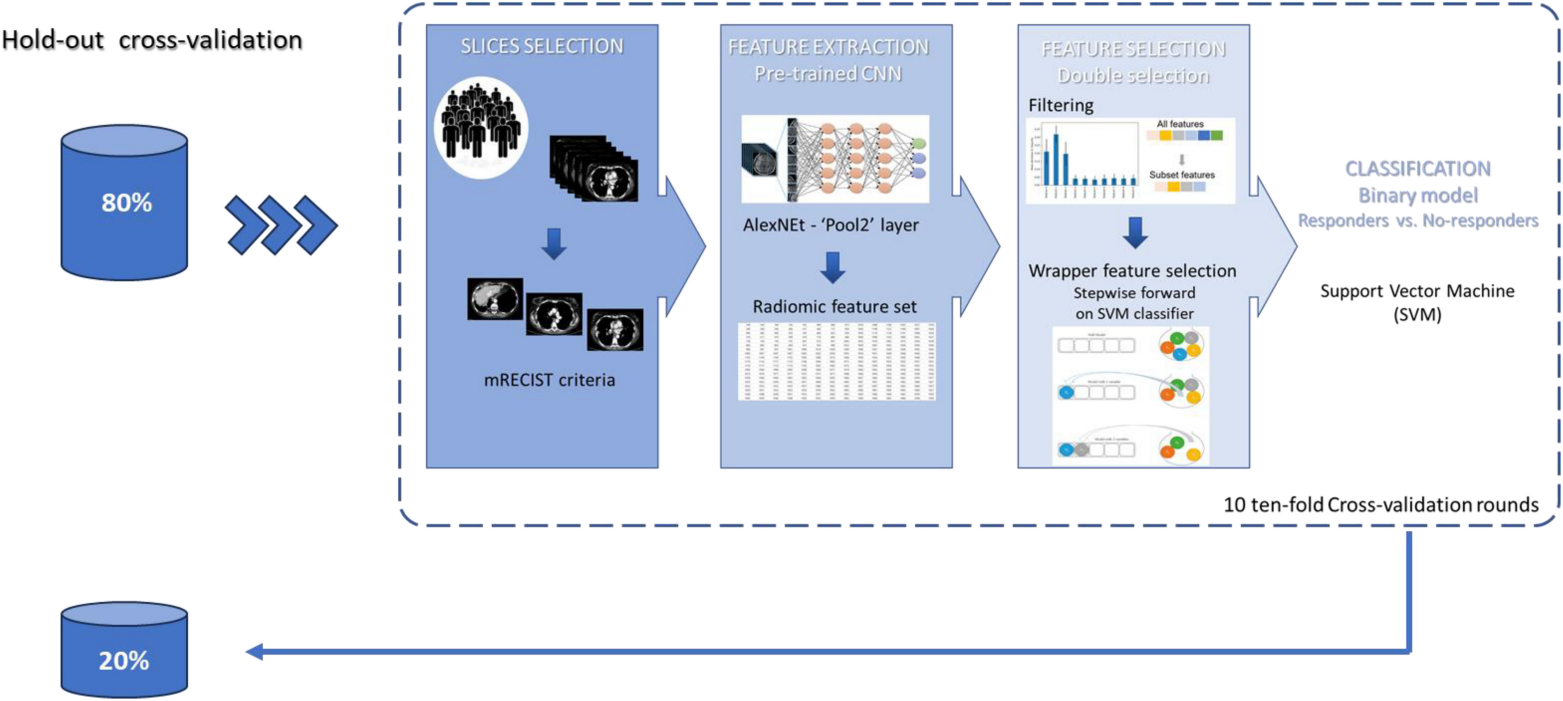
Analysis workflow. A set of radiomic features was extracted from the “Pool2” layer of AlexNet. A stepwise feature selection procedure was performed on the top 10 most discriminating features evaluated in terms of AUC. The model was evaluated with an 80:20 hold-out validation scheme. Moreover, the classification performances on the hold-out training set were evaluated in 10 tenfold validation rounds.

The purpose of this study was to predict the initial therapeutic response in patients with MPM. A machine learning model was trained to discriminate responders (partial response) from non-responders (stable or disease progression). **Figure 2** illustrates the overall pipeline of the classification model. For each patient, radiomic features were extracted from pre-trained CNN (as described in section 3.2). Then, a wrapper feature selection was performed on the top 10 most discriminating features evaluated in terms of AUC. Specifically, a stepwise feature selection procedure was implemented. Such a method follows a search approach of optimal features by evaluating different possible combinations of features according to a specific assessment criterion. In this study, we performed a forward feature selection based on the SVM classifier (46). The SVM classifier is a supervised machine learning algorithm that estimates the optimal hyperplane to separate the two classes by means of a kernel function. We used the linear function. The forward sequential selection algorithm identifies a subset of the features that best predict the expected result by sequentially adding at each step the feature that increases the performance of the SVM classifier evaluated in terms of AUC value on the training set of cross-validation. The procedure stops when 10 features have been included. Evaluations on a larger number of features (greater than 10) were made; however, the model did not improve performance. Indeed, also considering the small number of available patients, a greater number of features probably leads to an overfitting of the training set. The performance of the prediction classifiers was evaluated with an 80:20 hold-out validation scheme. Training and test hold-out sets were randomly selected by stratifying with respect to the outcome of interest. Moreover, the classification performances on the hold-out training set were evaluated in 10 tenfold validation rounds and expressed in terms of median and interquartile range (1st and 3rd quantile).

The classification metrics were evaluated in percentage terms of AUC value, and accuracy, sensitivity, and specificity were calculated by identifying the optimal threshold using Youden’s index on the ROC curves (46).

The same approach was used to determine the classification performances by shifting two slices up and down, with respect to those selected by our operator.

## 3 Results

### 3.1 Characteristics of dataset

**Table 1** summarizes the characteristics of patients. We have collected the following data: age, gender, comorbidities, previous asbestos exposure, ECOG performance status, body mass index (BMI), smoking habits with pack/year, histotype, disease stage, presence of pleural effusion, and type of first-line treatment.

A total of 38 patients with a median age at diagnosis of 70.27 (1st–3rd quartiles of 69.05–75.60) years afferent to our institute were studied. Among them, 25 patients (65.79%) showed an initial response to therapy, while stable or progressive disease was observed in the remaining patients.

### 3.2 Classification performances

As described in Materials and Methods, an SVM classifier algorithm was trained on the feature subsets selected by a double procedure of filtering and stepwise algorithm. The performance of the prediction classifiers was evaluated with 80:20 hold-out validation scheme and with 10 tenfold cross-validation rounds on the hold-out training set. Therefore, we split the sample of 38 patients into a training set of 30 patients and a test set of 8 patients used as an independent set.

Among the 30 patients in the training set, 20 had a partial initial response. The proposed model achieves the best performance when selecting seven features. **Table 2** summarizes the results reached by considering the CT slices selected by our operator. The model is highly performing with an AUC, accuracy, sensitivity, and specificity value of 78.50%, 80.00%, 87.50% and 75%, respectively.

**Table 2:** Classification performances achieved on the training and test set of the hold-out validation scheme by the radiomic-based model trained using the slices selected by our radiologist.

	Training test (30 pz.)	Test set (8 pz.)
Number of selected features	7	7
AUC (%)	78.50 (68.50–82.50)	86.67
Accuracy (%)	80.00 (76.67–86.67)	87.50
Sensitivity (%)	87.50 (80.00–95.00)	80.00
Specificity (%)	75.00 (70.00–80.00)	100

The median value and the interquartile range (1st and 3rd quantile) of AUC, accuracy, sensitivity, and specificity are reported in percentage values.

Among the eight patients in the test set, five had a partial initial response. The model achieved stable performances also on the test set. Indeed, our preliminary model has shown the values of median AUC value, accuracy, sensitivity, and specificity of 86.67%, 876.67%, 80.00%, and 100%, respectively.

The proposed model provides for the selection of the three reference slices by the radiologist according to the mRECIST criteria. We wanted to verify that the developed algorithm was robust with respect to variations in the selection of these slides. Therefore, we evaluated the model performances by shifting randomly two slices up or down in the pre-treatment CT images with respect to those identified by our operator. In this scenario, the number model selected four features and, as shown in **Table 3**, the model seems to remain highly performing even by varying the reference slices, by reaching an AUC value and an accuracy of 77.88% and 85.00% on the training set, whereas in the hold-out test set, they were 73.33% and 75.00%.

**Table 3:** Classification performance achieved on the training and test set of the hold-out validation scheme by the radiomic-based model trained using the shifted slices from those selected by our radiologist.

	Training test (30 pz.)	Test set (8 pz.)
Number of selected features	4	4
AUC (%)	77.88 (71.00–80.75)	73.33
Accuracy (%)	85.00 (80.00–86.67)	75.00
Sensitivity (%)	95.00 (85.00–95.00)	80.00
Specificity (%)	70.00 (50.00–70.00)	66.67

The median value and the interquartile range (1st and 3rd quantile) of AUC, accuracy, sensitivity, and specificity are reported in percentage values.

## 4 Discussion

Our experience aimed to evaluate the radiomic approach for predicting therapeutic response in patients with advanced/unresectable MPM. Some previous studies, mainly in other malignancies, showed interesting results by using radiomics to assess prognosis and predict the patients’ outcome (21, 23, 25–27, 29, 33, 35, 40–44).

Regarding MPM, in the last few years, the search for selecting some clinical and imaging-based biomarkers has grown (47, 48); given the recent availability of new therapeutic regimens, there is a need to better select subgroups of patients with different prognoses and levels of treatment responsiveness.

In particular, the microenvironmental landscape of mesothelioma and some clinical–biological determinants as circulating immune cells and cytokines or genomic characteristics could be integrated into histopathological aspects (49–58), thus better defining the prognosis and responsiveness to different therapeutic options. The AI techniques could be promising tools in the diagnostic and therapeutic management of pleural mesothelioma; indeed, the large amount of data that can be analyzed using these techniques could help to create algorithms potentially useful to the screening and/or early diagnosis of this disease as well as improve diagnostic accuracy through the introduction of digital pathology (59, 60). Based on previous studies that have highlighted the potential of radiomics, also in patients with MPM, this approach deserves further investigation.

It is noteworthy that the data extracted and processed by the AI techniques arise from imaging procedures, such as CT and PET scans, which are part of the diagnostic and therapeutic management of MPM according to clinical practice guidelines. Hence, such an approach could provide further information useful to MPM management without additional costs for the healthcare system and without any further and/or invasive diagnostic procedures.

The radiomics application to MPM has been generally investigated with the aim of improving the diagnosis and better defining the stage of disease (61–65). Our experience arose from the idea of testing whether radiomics and transfer learning techniques could be useful to assist clinicians in the choice of therapeutic strategy on the basis of a more accurate prognostic assessment and on the possible early prediction of the treatment outcome.

Currently, the recent expansion of the therapeutic landscape of MPM makes it necessary to select patients likely responsive to innovative therapies now available; therefore, the research of clinical and imaging data as biomarkers could support the treatment planning (66, 67).

Literature data about the possible identification of patients and disease characteristics of early prediction of treatment response are lacking and needed.

In this scenario, starting from the imaging performed routinely, the herein proposed tool may provide an early prediction of the initial response to therapy, helping to identify patients who will not respond after the first two cycles of therapy and who will probably deserve a different therapeutic management, or at least require a closer monitoring.

Although preliminary, our data about the initial response prediction are promising. The proposed model has been evaluated with cross-validation scheme reaching an AUC value and an accuracy of 78.50% and 80.00%, respectively. The classification performance was stable on the hold-out test set validation scheme with an AUC value and an accuracy of 86.67% and 87.50%, respectively. Furthermore, since the model was developed starting from the three CT slices selected by the radiologist according to the mRECIST criteria, as per clinical practice in the tumor staging phase, we wanted to evaluate how robust the model was to variations in the operator choice of slices of reference analyzed. By randomly shifting the slides indicated by our radiologist by two slices, the model proved to be stable.

Our model exploits CT images commonly carried out in clinical practice at baseline, so it fits right into this initial phase of the treatment process. The automated radiomic-based system here proposed aims to support clinicians in planning a tailored treatment strategy.

The main limitation of this study is the small number of patients, although the results refer to a homogeneous sample population, allowing to better highlight the association between the radiomic signature and the outcome of interest. Therefore, the future goal is the collation of a larger number of patients and with a longer follow-up. Moreover, in this work, we decided to use the entire slice of CT scan instead of a specific region of interest, given the particular radiological and morphological presentation patterns of pleural mesothelioma that could lead to evaluation bias in the segmentation phase. However, further insights of our study will also concern the evaluation of the model on suitably segmented regions of interest.

Moreover, in this study, we have preferred to use a shallower pre-trained neural network such as AlexNet. Compared to other networks that perform concatenation of operations before arriving at pooling (e.g., ResNet or DenceNet class), AlexNet learns features that are more generalizable by increasing the classification capacity of the medical image database. However, in future studies, we will evaluate and compare other pre-trained CNN architectures (68). Finally, since these are radiomic features extracted from a pre-trained convolutional network, and not handcrafted features, it is not possible to explain the meaning of the selected features. Indeed, unlike handcrafted features for which the physical meaning of the extracted radiomic features is known, it is particularly complex to explain the meaning of radiomic features extracted from a pre-trained convolutional network because it is extracted on images that have undergone a series of convolutional operations, pooling, and normalization. However, the dense literature on biomedical image analysis has amply demonstrated that features extracted through pre-trained CNNs are more informative than handcrafted ones because they capture the local structure of the image at a deeper level (points, edges, textures, etc.). However, future developments of the study involve collecting a significantly larger study sample that will enable end-to-end training of a CNN. In that case, as the features are extracted directly from the image of interest and not by transfer of knowledge from a pre-trained network of a different nature (transfer learning), it is possible to identify at least the areas found to be most informative through the application of recent explainable AI techniques (69, 70).

A further future development, in the perspective of defining a highly personalized model, could be a multimodal model that can also jointly evaluate, in addition to radiomic characteristics, quantitative characteristics extracted from images of digitalized biopsy slides and genomic data. Certainly, it is a particularly ambitious project that requires a significant effort to collect a consistent and complete case history of information and data, especially given the rare nature of the pathology under examination. Nonetheless, the development of such a multimodal model could lead to valuable results useful for patient management.

## 5 Conclusions

The lack of screening tests, albeit for subjects at risk due to previous asbestos exposure, together with the generally late diagnosis due to the absence of specific symptoms, makes Malignant pleural mesothelioma a tumor with a poor prognosis. For this reason, AI approaches for a better definition of prognostic models assume an important role in modern medicine in general, even more so for rare pathologies such as the one object of this work. Our proposed approach is particularly valuable as it aims to provide these insights without the need for invasive procedures or incurring additional costs beyond those typically associated with standard clinical practice.

In addition, our model could represent a part of a multimodal strategy by including it in a clinical workflow not only to improve the prognostic assessment of patients with MPM but also to identify subgroups of patients with different responsiveness to treatments. A further qualifying element of this approach is represented by the possibility of using AI techniques based on CT scans and images normally used in clinical practice, therefore not requiring invasive or additional tests for patients. Although further validation on a larger sample population is needed, our study supports the hypothesis that AI techniques, by evaluating radiomics and other parameters (clinical, pathological, and genomic data), could generate algorithms potentially useful to predict patient outcomes. Such a global approach deserves further investigation given that innovative treatments are being studied and are about to become more widely available in MPM; in this scenario, the choice of the treatment strategy should consider the costs of new drugs and the need to avoid unnecessary toxicities, thus preserving the quality of life of unresponsive patients and finally leaning towards the goal of precision medicine in patients with MPM as well.

## Data Availability

The raw data supporting the conclusions of this article will be made available by the authors, without undue reservation.
